# The Role of Genetic and Immune Factors for the Pathogenesis of Primary Sclerosing Cholangitis in Childhood

**DOI:** 10.1155/2016/3905240

**Published:** 2016-11-02

**Authors:** Priscila Menezes Ferri, Ana Cristina Simões e Silva, Soraya Luiza Campos Silva, Diego Junior Queiroga de Aquino, Eleonora Druve Tavares Fagundes, Débora Marques de Miranda, Alexandre Rodrigues Ferreira

**Affiliations:** ^1^Department of Pediatrics, UFMG, 30130-100 Belo Horizonte, MG, Brazil; ^2^Instituto Nacional de Ciência e Tecnologia de Medicina Molecular, INCT-MM, CNPq-FAPEMIG, Universidade Federal de Minas Gerais, 30130-100 Belo Horizonte, MG, Brazil; ^3^Laboratório Interdisciplinar de Investigação Médica, Avenida Alfredo Balena 190, 2nd Floor, Room 281, 30130-100 Belo Horizonte, MG, Brazil

## Abstract

Primary sclerosing cholangitis (PSC) is a rare cholestatic liver disease characterized by chronic inflammation of the biliary tree resulting in liver fibrosis. PSC is more common in male less than 40 years of age. The diagnosis of PSC is based on clinical, laboratory, image, and histological findings. A biochemical profile of mild to severe chronic cholestasis can be observed. Endoscopic retrograde cholangiography is the golden standard method for diagnosis, but magnetic resonance cholangiography is currently also considered a first-line method of investigation. Differences in clinical and laboratory findings were observed in young patients, including higher incidence of overlap syndromes, mostly with autoimmune hepatitis, higher serum levels of aminotransferases and gamma-glutamyl transferase, and lower incidence of serious complications as cholangiocarcinoma. In spite of the detection of several HLA variants as associated factors in large multicenter cohorts of adult patients, the exact role and pathways of these susceptibility genes remain to be determined in pediatric population. In addition, the literature supports a role for an altered immune response to pathogens in the pathogenesis of PSC. This phenomenon contributes to abnormal immune system activation and perpetuation of the inflammatory process. In this article, we review the role of immune and genetic factors in the pathogenesis of PSC in pediatric patients.

## 1. Introduction

Autoimmune liver diseases are infrequent in children and adolescents, but, when diagnosed, prompt treatment has utmost importance. Among these conditions, primary sclerosing cholangitis (PSC) is a rare cholestatic liver disease characterized by chronic inflammation of the biliary tree resulting in liver fibrosis. An important factor for the occurrence of cirrhosis is the toxic effect of bile stasis [1, 2]. In contrast with the female predominance of many autoimmune diseases, approximately 2/3 of the PSC patients are males aged less than 40 years [3].

PSC has a wide-ranging clinical presentation, from asymptomatic patients to chronic liver disease [4, 5]. As a result, the diagnosis can be a big challenge, especially in young patients. Cholestatic bile diseases are usually associated with other autoimmune conditions, including ulcerative colitis (UC) and autoimmune hepatitis, referred to as the overlap syndromes [6, 7]. The named AIH-PSC syndrome is more frequent in children and adolescents than in adult patients; the disease evolves with typical features of PSC in cholagiography or histology, associated with laboratory and histological characteristics of AIH. In AIH-PSC, the reaction to treatment is also different, since patients have better response to immunosuppression than the isolated form of PSC [7].

The diagnosis of PSC is based on clinical, laboratory, and image findings. A biochemical profile of mild to severe chronic cholestasis can be observed [8]. Endoscopic retrograde cholangiography is the golden standard method to detect PSC. The exam shows multifocal areas of strictures of intra- and/or extrahepatic bile ducts, with intervening segments of normal or dilated ducts [8, 9]. In addition, magnetic resonance cholangiography (MRC) is also considered a first-line image method for PSC diagnosis, being noninvasive and reliable [8, 10, 11].

Multiple autoantibodies can be useful in detecting PSC, but none of them solely allows the diagnosis [12]. Serum atypical perinuclear antineutrophil cytoplasmic antibody (p-ANCA) is the most common autoantibody detected in PSC, but with a weak association [13–15]. The second commonest autoantibodies are anti-*Saccharomyces cerevisiae* antibodies (ASCA), which also exhibit high frequency even in the absence of advanced disease or inflammatory bowel disease (IBD) [16]. Recently, Jendrek et al. [17] also found an association between antibodies against glycoprotein 2 (anti-GP2) and large bile duct diseases with considerable rates. In PSC, anti-GP2 IgA was consistently identified in patients with poor survival during follow-up, being associated with cholangiocarcinoma [17]. Other autoantibodies including antinuclear antibody (ANA) and liver kidney microsomal type 1 antibody (anti-LKM1) can be positive in PSC patients, but with low specificity for the diagnosis of the disease [18].

PSC has no effective medical treatment and, in many cases, the disease will lead to cirrhosis and end-stage liver disease with need of liver transplantation [1, 5, 19, 20]. Ursodeoxycholic acid (UDCA) has been administered as a palliative measure, without interfering with clinical outcome [21, 22]. Since clinical tools have been insufficient to characterize and to predict the outcome, the aim of this review is to summarize evidence from literature about the potential role of immune and genetic factors in the pathogenesis of PSC in pediatric patients.

## 2. Genetic Factors

The pathogenesis of PSC is still not fully understood, but a complex interaction between genetic, immunological, and environmental factors with breakdown of self-tolerance has been reported [14, 23]. Studies have shown a strong genetic predisposition in PSC, with first-degree relatives exhibiting 9- to 39-fold increased risk to develop the disease [24]. Genome studies showed that this genetic tendency is mainly associated with human leukocyte antigen (HLA) complex II (MHC II) chromosome 6p21 [25–29]. Some haplotypes are considered as main susceptibility factors. HLA-B and HLA-DRB1 alleles are the most important ones. Among them, the most frequent are HLA-A1-B8-DRB1*∗*0301-DQB1*∗*0201, HLADRB1*∗*1301-DCB1*∗*0603, and HLA-DRB1*∗*1501-DQB1*∗*0602 [25–27, 30, 31]. As protective haplotypes, HLA-DRB1*∗*04-DQB1*∗*0302 and HLA-DRB1*∗*0701-DQB1*∗*0303 have been reported [25, 26, 31]. In spite of the detection of several HLA variants as associated factors in large multicenter cohorts of adult patients, the exact role and pathways of these susceptibility genes remain to be determined in pediatric population [25–29].

Table 1 is a compilation of relevant studies that have investigated the influence of MHC II antigens in PSC. The number of children evaluated in these studies is also shown.

Other studies have proposed the existence of MHC class I region genes and non-HLA risk loci for PSC, supporting that other genetic factors take part in the pathogenesis of the disease. Wiencke et al. [43] evaluated the extended HLA class I region genes as contributing factors to modulate immune response or to confer susceptibility in PSC patients. The authors found a significant association with alleles MIB*∗*349, D6S265*∗*122, D6S464*∗*209, and D6S2225*∗*147, all being secondary to DR3 (HLA-DRB1*∗*03) associations [43]. D6S265*∗*122 was also associated with DR6 [40]. The study of Karlsen et al. [44] showed that killer immunoglobulin-like receptors (KIRs) and HLA class I ligands can be also associated with PSC.

Norris et al. [45] described an association between the* MICA∗008* allele and PSC.* MICA∗008* is one of the components of a group of polymorphic genes on chromosome 6.* MICA∗008* is a stress and heat shock inducible molecule that activates inflammatory response as a ligand for *γδ* T cells, natural killer (NK) (CD56^+^) cells, and cells expressing the NKG2D receptor [42].

Non-HLA findings include modifications in genes related to autoimmunity (IL2/IL2RA), bile acid toxicity (GPBAR1), and mechanisms related to concomitant IBD (IL2/IL21, ILR2A, CARD9, MST1, Fut2, and SIK2) [27, 46]. Karlsen et al. [25] also investigated genome-wide association in PSC. These authors detected a susceptibility variant of importance for inflammatory conditions at chromosome* 13q31*, with GPC5/GPC6 as a candidate to this association [25]. Despite its involvement in inflammatory pathways, the precise function of GPC6 in the liver and bile ducts remains unknown and more studies are needed to clarify the role of this molecule.

As shown, most of these studies evaluated only adults, creating a gap in relation to the findings of pediatric patients. Indeed, PSC in children seems to be different from the disease in adults [44, 45]. In this regard, differences in clinical and laboratory findings observed in young patients include higher incidence of overlap syndrome, higher serum levels of aminotransferases and gamma-glutamyl transferase, and lower incidence of serious complications as cholangiocarcinoma [44]. Thus, the results obtained for adult patients may not be always valid for children. Further studies are obviously needed to evaluate the role of immune and genetic factors in pediatric age group. Studies should analyze pediatric patients as a subgroup by separating patients who had disease onset during childhood. This strategy of analysis probably enables more reliable evaluation of the role of immune and genetic factors in pediatric PSC.

## 3. Immune Factors

The literature supports a role for an altered immune response to pathogens in the pathogenesis of PSC [14, 47]. This phenomenon contributes to abnormal immune system activation and perpetuation of an inflammatory process [14, 47]. Cholangiocytes, after antigenic stimulus, release proinflammatory mediators that stimulate immune cells. The interaction between toll-like receptors (TLRs) and pattern-associated molecular patterns (PAMPs) promotes a persistent inflammatory environment for cholangiocytes [48, 49]. TLR activation can increase the expression of interleukin-6 (IL-6) and of cluster for differentiation 44 (CD44) that acts as an adhesion molecule for lymphocytes [48]. Along with this process, tumor necrosis factor (TNF), IL-6, and IL-8, released by cholangiocytes and immune cells, trigger the recruitment and activation of T lymphocytes, macrophages, neutrophils, NK, and mesenchymal cells [14, 50–53]. In addition, senescence has been recently shown to be an important pathologic process in diverse conditions, since senescence cells can be associated with proinflammatory cytokine and chemokine hypersecretion, referred to as a senescence associated secretory phenotype (SASP) [54]. In this regard, Tabibian et al. [55] showed that PSC cholangiocytes present higher expression of SASP components, including IL-6, IL-8, CCL2, and PAI-1. These cells also had increased expression of N-Ras, a well-known inducer of senescence. These findings support the role of senescence in PSC.

An increased number of activated lymphocytes and of NK cells in peripheral blood of PSC patients were also detected [53, 56]. However, Bo et al. [57] showed that T lymphocytes function is impaired in PSC patients when compared with health controls. In regard to NK cells, it was reported that these cells are activated by lipid antigens exposed by the MHC class I-like molecule, CD1d [58]. When activated, NK cells can play either a protective or detrimental role in autoimmune diseases [58]. In a recent study, Schrumpf et al. [59] reported that human cholangiocytes can express CD1d and that CD1d is downregulated in biliary epithelium of patients with PSC. These authors also showed that cholangiocytes unmask lipid antigens to NL cells, suggesting that this mechanism could play a role in the autoreactivity of NK cells in PSC [59].

There are some hypotheses about how cytokines and adhesion molecules may contribute to PSC pathogenesis. In this context, lymphocytes express specific receptors, *α*4*β*7 and CCR9, with these cells being responsible for interferon-*γ* (IFN-*γ*) production, which, in turn, enhances inflammatory stimuli [60, 61]. Additionally, due to the release of cytokines as CCL28, CXCL12, and CXCL16 and the presence of activated lymphocytes,* naive* T cells can be primed to T helper 1-cells [62, 63]. In line with these findings, Martins et al. [64] showed that *γδ* ^+^ cells expressing CD45RO and IL-2 contribute to an activated memory mechanism that maintains the inflammatory process. Sebode et al. [65] had also described a reduction of regulatory immune cells, including FOXP3^+^ cells and T reg cells, in PSC patients. Indeed, an increased production of inflammatory cytokines including IL-17, IL-21, and TNF and of IL-17A-expressing lymphocytes can be found around bile ducts [66]. In this regard, Liu et al. [29] showed novel loci related to cytokines and chemokines in patients with PSC. The authors detected six loci in which the same gene was found by more than one technique, supporting the role of these genes in PSC [29]. At position 11q23, the most strongly associated single nucleotide polymorphism (SNP), rs7937682, is located in an intron of* SIK2* gene, which encodes salt-inducible kinase 2. This protein influences the expression of both IL-10 in macrophages and Nur77, an important transcription factor in leukocytes [29]. Another relevant association was found at 12q13, which is an intronic SNP, rs11168249, within the* HDAC7* gene, that encodes histone deacetylase 7 [29]. This molecule has been implicated in the negative selection of T cells in the thymus, a key process in the development of immune tolerance. More studies are needed to elucidate the clinical value of these genetic findings and whether these genes can also be related to pediatric PSC.

The CCR5 (chemokine receptor 5) is responsible for the recruitment of activated lymphocytes via portal expression of CCR5 ligands [67, 68]. CCR5 also contributes to generation of T helper 1 immune response [68, 69]. Despite controversial findings, some studies report that CCR5-Delta32 deletion is associated with significant reduction in cell surface expression of this chemokine receptor, thus compromising lymphocytes activation [67–69]. While Eri et al. [68] showed that CCR5-Delta32 allele frequency was significantly higher in PSC compared to controls, Melum et al. [69] did not find any statistically significant difference in the occurrence of this mutation in patients and controls.

Some studies showed that mucosal addressin cell adhesion molecule (MAdCAM-1) plays an important role in T-lymphocyte recruitment to liver tissue derived from the gut by the connection with *β*2-integrin ligand [61, 67, 70]. This mechanism explains the hepatic recruitment of mucosal lymphocytes in inflammatory liver diseases. For patients with IBD and associated PSC, this pathway has been considered as a potential therapeutic target [71]. There are studies evaluating antiadhesion molecule therapies, but yet without convincing results [71].

Liaskou et al. [72] showed that when compared with CD28^+^ T cells, activated CD28^−^ T cells produce high levels of IFN*γ* and TNF, leading to upregulation of intercellular cell adhesion molecule-1, HLA-DR, and CD40, which are important ligands for immune activated T cells. The authors also described significantly greater proportion of CD4^+^CD28^−^ and CD8^+^CD28^−^ cells that infiltrate in liver tissue of patients with PSC, leading to a proinflammatory environment rich in TNF [72].

## 4. Concluding Remarks

In conclusion, HLA class I and class II are shown to be the main risk factors for PSC in the MHC, but it is time to consolidate available information and to translate research findings into applicable knowledge for clinical practice. Studies with children are infrequent, and the findings obtained in adults should not be extrapolated for this age group. Changes in immune response to pathogens, activation of T lymphocytes, and release of inflammatory and adhesion molecules also contribute to the pathogenesis of PSC. More studies are clearly needed to unveil the influence of genetic and immune factors in the pathogenesis of PSC in pediatric patients and how these markers can be used as diagnostic tools and/or therapeutic targets.

## Figures and Tables

**Table 1:** Publications on major histocompatibility class II human leukocyte antigens and their association with primary sclerosing cholangitis patients.

Reference	Total number of patients/controls (number of children and adolescents)	What was evaluated	Conclusions
Farrant et al. [32], 1992	71/68 (0)	HLADRB, DQA, and DQB	HLADRB3*∗*0101 was the most associated allele, with reduced survival of patients with it. DRB5*∗*0101 was another susceptibility allele and DRB4*∗*0101 demonstrated a likely protective function.

Amar et al. [33], 1992	15/no control (0)	HLADRB3	No apparent association of the alleles of the DRB3 locus in the Israeli population.

Olerup et al. [34], 1995	75/250 (not cited)	HLADR and DQ	Association with DRB1*∗*1301, DQAI*∗*0103, and DQBI*∗*0603 haplotype was confirmed, whereas DRB1*∗*04 was only slightly underrepresented. No difference was observed in age, presentation, liver function, histological stage, or survival between patients with different positive alleles.

Leidenius et al. [35], 1995	24/106 (not cited)	HLA-A, B, C and DR	HLA-B8 and DR3 (DRB1*∗*03) were associated with primary sclerosing cholangitis.

Wilschanski et al. [36], 1995	27/no control (all children)	HLA-B and HLADR	An increased incidence of HLA B8 and DR2 (DRB1*∗*15) but not DRw52a (DRB3*∗*0101) was found.

Spurkland et al. [30], 1999	256/764 (not cited)	HLADR and DQ	Increased frequencies of DRB1*∗*03-DQA1*∗*0501-DQB1*∗*02, DRB1*∗*13-DQA1*∗*0103-DQB1*∗*0603, and DRB1*∗*15-DQA1*∗*0102-DQB1*∗*0602 haplotypes were observed. PSC was negatively associated with DRB1*∗*04-DQB1*∗*0302 haplotype.

Boberg et al. [37], 2001	265/no control (yes, but the number was not cited)	HLADR and DQ	DRB1*∗*03-DQA1*∗*0501-DQB1*∗*02 (i.e., DR3, DQ2) heterozygous genotype was associated with an increased risk of death or liver transplantation. Presence of a DQ6 encoding haplotype (DQB1*∗*0603 or DQB1*∗*0602) in DR3, DQ2 negative individuals was associated with a reduced risk of death or liver transplantation.

Donaldson and Norris [31], 2002	148/134 (0)	HLADR and DQ	Associations with the DRB3*∗*0101-DRB1*∗*0301-DQA1*∗*0501-DQB1*∗*0201 and DRB1*∗*1301-DQA1*∗*0103-DQB1*∗*0603 haplotypes were confirmed. Protective influence of the DRB1*∗*04-DQB1*∗*0302 haplotype was reaffirmed. A previously unreported protective haplotype was found: DRB1*∗*0701-DQB1*∗*0303.

Bittencourt et al. [38], 2002	63/83 (27)	HLA-B, DRB1, DQB1	No increase in the frequency of HLA-B, DRB3, DRB4, or DRB5 alleles was observed. The frequency of HLA-DRB1*∗*1301 and HLA-DQB1*∗*06 was significantly increased in PSC patients.

Neri et al. [39], 2003	64/183 (0)	HLA-DRB1, HLA-DQB1, and HLA-B	Frequencies of DRB1*∗*01-DQA1*∗*0101-DQB1*∗*0102, DRB1*∗*16-DQA1*∗*0102-DQB1*∗*0502, and DRB1*∗*04-DQA1*∗*03-DQB1*∗*0301 haplotypes were more elevated in PSC patients. DRB1*∗*07-DQA1*∗*0201-DQB1*∗*02 haplotype frequency was significantly decreased in patients.

Karlsen et al. [25], 2010	285/298 (yes, but the number was not cited)	HLADR and DQ	The strongest association was detected for HLA-B*∗*08 and associations with the DRB1 alleles -DRB1*∗*03, -DRB1*∗*04, -DRB1*∗*07, and -DRB1*∗*1301 also were confirmed.

Hov et al. [40], 2010	78/79 (not cited)	HLADRB1, HLA-C	Positive association of PSC with HLADRB1*∗*15, -DRB1*∗*03, -DRB1*∗*04, and -DRB1*∗*1301 was confirmed. A protective association with HLADRB1*∗*0701 was found.

Wang et al. [41], 2014	31/42 (all children)	HLADR haplotypes	Frequencies of homozygous HLA DRB1*∗*0301 (DR3) genes and haplotype A1-B8-DR3 were higher in patients. Frequencies of disease-protective genes DR4 and/or DR15 were lower in the patients.

Næss et al. [42], 2014	365/368 (yes, but the number was not cited)		HLADRB1*∗*1301-DQB1*∗*0603 and DRB1*∗*1501 haplotypes conferred risk for PSC. HLADRB1*∗*04-DQB1*∗*03, DRB1*∗*0701-DQ*∗*0303, and DR13:XX (all non-13:01 alleles)-DQB1*∗*06 demonstrated a protective effect.

HLA: human leukocyte antigen; MHC: major histocompatibility complex; PSC: primary sclerosing cholangitis.
